# Squalamine and claramine A1 disperse *Pseudomonas aeruginosa* biofilm

**DOI:** 10.1016/j.bioflm.2025.100293

**Published:** 2025-05-27

**Authors:** Anne-Sophie Tareau, Ali Tahrioui, Mathieu Gonzalez, Evan Croize, Jennifer Varin Simon, Magalie Barreau, Audrey David, Fany Reffuveille, Jean-Michel Brunel, Olivier Lesouhaitier, Sylvie Chevalier

**Affiliations:** a Univ Rouen Normandie, Université Caen Normandie, Normandie Univ, CBSA UR 4312, F-76000, Rouen, France; b Univ Rouen Normandie, Platform of Sanitary Safety, Evreux Campus, PS2E, France; c Université de Reims Champagne-Ardenne, BIOS, Reims, France; d Aix Marseille Univ, INSERM, SSA, MCT, F-13385, Marseille, France

**Keywords:** *Pseudomonas aeruginosa*, Biofilm, Eradication, Squalamine, Claramine A1, Spermine, Spermidine

## Abstract

*Pseudomonas aeruginosa* is an opportunistic pathogen that causes both acute and chronic infections, including pneumonia, bloodstream infections, urinary tract infections, and surgical site infections. It poses a significant threat to individuals with chronic lung conditions, particularly those with cystic fibrosis. Squalamine and claramine A1 have emerged as promising antibacterial compounds, exhibiting activity against a broad range of both Gram-positive and Gram-negative bacteria. Beyond their potent antibacterial properties, our findings reveal that sub-inhibitory concentrations of claramine A1 and squalamine can disperse pre-formed *P. aeruginosa* biofilm without impacting bacterial growth. While claramine A1, but not squalamine, enhances membrane fluidity, the structural difference between these compounds lies primarily in their spermine and spermidine moieties. Notably, we found that spermine, unlike spermidine, was able to both disperse biofilm and increase membrane fluidity. Together, our results suggest that while both compounds are effective at disrupting *P. aeruginosa* biofilm, they likely act through distinct mechanisms.

## 1 Introduction

**Fig. 1:**
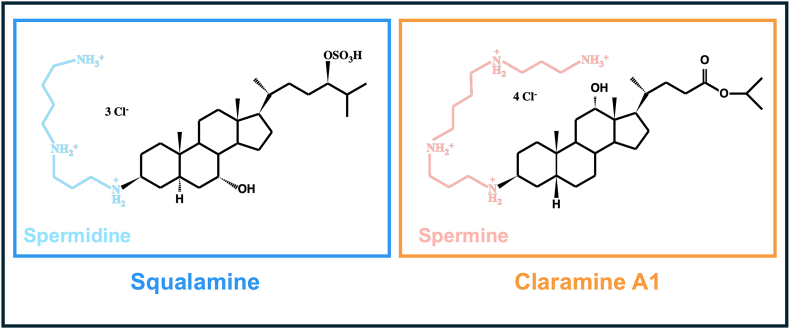
Chemical structures of squalamine and claramine A1, with their spermidine and spermine moieties, highlighted in blue and orange, respectively.

*Pseudomonas aeruginosa* is an opportunistic pathogen responsible for both acute and chronic infections, including pneumonia, bloodstream infections, urinary tract infections, and surgical site infections. It is particularly dangerous for immunocompromised individuals with chronic lung diseases, especially those with cystic fibrosis. In addition, some bacterial strains have acquired resistance to the last-line of carbapenem antibiotics, leading the World Health Organization to classify *P. aeruginosa* as a priority for the discovery of new antibiotics [1,2]. *P. aeruginosa* also becomes highly tolerant to antibiotics when grown within biofilms. Bacterial biofilms are communities aggregated within a self-produced extracellular matrix composed of exopolysaccharides, proteins, lipids, nucleic acids, and outer membrane vesicles [3]. This matrix enables cell cohesion and surface attachment and allows the biofilm scaffold formation and bacterial protection from exogenous aggressions, including antibiotics and immune system-related effectors [4]. In particular, some biofilm-enclosed bacterial subpopulations are up to 1000-fold more tolerant to antibiotics than planktonic cells [3,5]. These biofilms play a critical role in the pathogenesis of most chronic infections in humans, whether tissue-specific or associated with medical implants [6]. Biofilm-associated infections are notably resistant to host immune responses and often trigger excessive or dysregulated inflammation, leading to further tissue damage and the spread of infection [7]. Moreover, these biofilms demonstrate remarkable resilience against antimicrobial therapies [8]. Given these properties that attribute a chronic character to such infections, there is an urgent need for new antibiotics and/or therapeutic strategies to combat *P. aeruginosa*, particularly in its biofilm-associated form. Squalamine and aminosterols have emerged as a promising family of antibacterial compounds. Due to the impracticality of large-scale extraction of squalamine from its natural source, the dogfish shark [9,10], and the complexity of its chemical synthesis—which involves multiple steps and yields low product quantities [11,12]—, researchers have developed squalamine mimics and explored their antimicrobial properties [13,14]. Squalamine is a cationic aminosterol that exhibits a broad-spectrum antibiotic activity against both Gram-positive and Gram-negative bacteria. It also potentiates the activity of several antibiotic classes at sub-inhibitory concentrations [15]. This compound consists of a sterol core with a sulfated side chain and a hydrophilic polyamine spermidine moiety attached to the hydrophobic unit (Fig. 1). Squalamine is a membrane-active molecule that disrupts the outer membrane of Gram-negative bacteria *via* a detergent-like mechanism. Its positively charged amino groups facilitate binding to negatively charged lipopolysaccharides in a calcium ion-dependent manner ([16]; Di Pasquale et al., 2010). Squalamine was also shown to depolarize the cytoplasmic bacterial membranes [16,17]. Noticeably, either the sterol, the spermidine chain, or the sulfate group, were shown to display only a low effect on their antimicrobial activity alone [18,19]. Recently, the spermidine group of the squalamine was suggested to interact with the accumulation-associated protein Aap of the surface of the human skin commensal *Staphylococcus epidermidis*, allowing the initial binding drug to the cell wall [18]. In addition, squalamine was shown to inhibit the glycosyltransferase (GTase) activity of the peptidoglycan glycosyltransferase PBP1b of *Escherichia coli* by an unknown mechanism [20]. Recently, Blanchet et al. reported the design and biological evaluation of claramine A1, a water-soluble compound that is synthetically accessible and exhibits antimicrobial activity against a broad spectrum of Gram-positive and Gram-negative bacteria, including antibiotic-resistant strains [21]. Claramine A1 was synthesized from a deoxycholic acid derivative in just three steps, involving a key reductive amination with spermine at the C6 position (Fig. 1; [21]).Claramine A1 was proposed to act *via* two ways on *Enterobacter aerogenes* EA289, *i.e*., through disruption of the outer membrane as previously described for squalamine, and *via* inhibition of the AcrAB-TolC efflux pump through disruption of the transmembrane potential [21]. Interestingly, in addition to their large antibacterial activity [22], squalamine and some of its 6-aminosterol-derivatives were shown to affect biofilm when grown in microtiter plates [23].

Herein, we demonstrate that sub-inhibitory concentrations of claramine A1 and squalamine can disperse pre-formed *P. aeruginosa* biofilms without inhibiting bacterial growth. Claramine A1 was found to increase membrane fluidity, at least in part by activating the cell envelope stress response, whereas squalamine did not exhibit these effects. The structural difference between claramine A1 and squalamine relies mainly on their spermine or spermidine moieties, respectively. Interestingly, we show that spermine was able to disperse biofilm and enhance membrane fluidity, while spermidine did not. Altogether, our data suggest that both molecules, while efficient in dispersing *P. aeruginosa* biofilm, would act through different pathways.

## 2 Results

### 2.1 Screening of squalamine and claramine A1 for their effects on *P. aeruginosa* planktonic growth, biofilm formation and dispersion

**Fig. 2:**
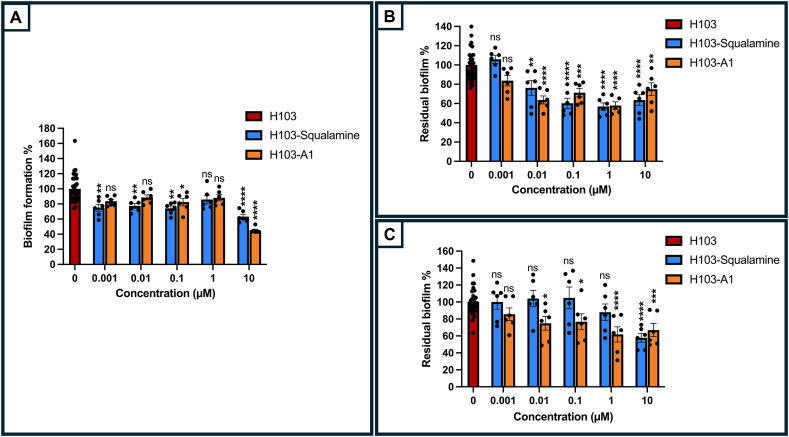
Squalamine (blue bars) and claramine A1 (orange bars) can prevent biofilm formation (A) and eradicate a 24 h-preformed biofilm upon 2 h (B) or 24 h (C) treatment at different concentrations. The error bars represent the standard error of the means (SEMs) and are the result of the analysis of three independent biological assays. Statistics were achieved by ordinary one-way ANOVA followed by Tukey's multiple-comparison test. Significance was considered at ∗∗∗∗, *p* < 0.0001; ∗∗∗, *p* = 0.0001–0.001; ∗∗, *p* = 0.001–0.01; ∗, *p* = 0.01–0.05; ns (not significant), p > 0.05.

Both compounds have been evaluated for their antibacterial activity under our experimental conditions, leading to the determination of their Minimal Inhibitory Concentrations (MICs). In LB medium, both squalamine and claramine A1 exhibited minimum inhibitory concentrations (MICs) of 16 μg mL^−1^, corresponding to 25.48 μM and 25.85 μM, respectively (Supplementary Fig. S1). To investigate the effects of sub-MIC, serial 1:10 dilutions ranging from 0.001 μM to 10 μM were tested on *P. aeruginosa* H103, assessing their impact on planktonic growth, biofilm formation, and biofilm dispersion. As shown in Supplementary Fig. S2, squalamine did not affect *P. aeruginosa* H103 growth at each of the tested concentrations. The same result was observed for claramine A1, except when used at 10 μM, for which the growth kinetic was lowered (Supplementary Fig. S2). Biofilm formation assay is relevant to search for compounds that are capable of preventing biofilm initiation and development. Herein, squalamine, claramine A1, or ultrapure Milli-Q® water (control), were added to LB at the beginning of the assay. In these conditions, squalamine led to reduce biofilm formation by about 25 % from 1 to 0.001 μM, and 37 % at 10 μM (Fig. 2A). Claramine A1 shows significant inhibition effects at 10 μM by about 55 % (Fig. 2A). Biofilm dispersion is relevant to search for compounds that can eradicate an existing biofilm and was initially investigated in a first attempt using Calgary devices. A 24 h-preformed biofilm grown on pegs was treated with a concentration range between 0.001 μM and 10 μM of squalamine, or claramine A1 for 2 h (Fig. 2B), or for 24 additional hours (Fig. 2C), followed by crystal violet staining. As shown in Fig. 2B, both compounds led to disperse a 24 h-preformed *P. aeruginosa* biofilm, with a maximal effect at 1 μM of about 42 %, upon a 2 h treatment. Noticeably, biofilm eradication was shown at concentrations as low as 0.01 μM with squalamine or claramine A1. Biofilm dispersion was also shown after 24 h of treatment with both compounds but to a lesser extent, the maximal dispersal effect was observed at 10 μM for squalamine, and at 1 and 10 μM for claramine A1 (Fig. 2C). While claramine A1 displays also a dispersal effect of about 25 % at 0.01 μM, no impact was shown for squalamine at concentrations below 10 μM, suggesting that claramine A1 is more efficient for biofilm disruption in this latter condition (Fig. 2C). In this study, we focus on the biofilm dispersion phenotype upon 2 h treatment of squalamine or claramine A1 at 1 μM in the subsequent assays, to gain further insights into the related mechanism. The sessile and planktonic lifestyles are known to be inversely regulated, with the planktonic lifestyle being associated with the production of virulence factors [24]. We then investigated the effect of both squalamine and claramine A1 at 1 μM each, on the production of pyocyanin, a major virulence factor of *P. aeruginosa*. As shown in Supplementary Fig. S3, none of these two aminosterols modulate the production of pyocyanin in our conditions.

### 2.2 Squalamine and claramine A1 disperse *P. aeruginosa* biofilms

**Fig. 3:**
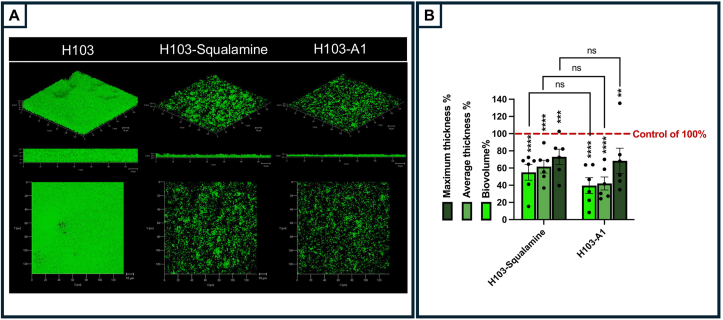
Squalamine and claramine A1 can disperse a 24 h-preformed biofilm of *P. aeruginosa* H103. (A) 3D shadow (upper panel), side (middle panel), and top (lower panel) CLSM images representative of a 24 h-old biofilm of *P. aeruginosa* after bacterial labeling with SYTO 9 (green) before (H103) and after 2 h of treatment with squalamine (H103-squalamine) or claramine A1 (H103-A1) at 1 μM concentration. Images show representative data from six independent biofilm assays. (B) COMSTAT2 analyses were performed to determine maximum thickness, average thickness and biovolume of the control condition (*P. aeruginosa* H103 treated with ultrapure Milli-Q® water for 2 h), and the assays (*P. aeruginosa* H103 upon squalamine or claramine A1 treatment for 2 h). The control parameters were put at 100 % (red line). The error bars represent the standard error of the means (SEMs) and are the result of the analysis of at least five views of each of the six independent biological assays. Statistics were achieved by ordinary one-way ANOVA followed by Tukey's multiple-comparison test. Significance was considered at ∗∗∗∗, *p* < 0.0001; ∗∗∗, *p* = 0.0001–0.001; ∗∗, *p* = 0.001–0.01; ns (not significant), p > 0.05.

To further validate these findings, the anti-biofilm effects of squalamine and claramine A1 were assessed using confocal laser scanning microscopy (CLSM), allowing for more precise quantification of the remaining biomass upon exposure to both compounds. Biofilms of *P. aeruginosa* H103, grown for 24 h, were treated with 1 μM of squalamine or claramine A1 for 2 h before being labeled with SYTO® 9 green fluorescent nucleic acid stain and observed by CLSM. As a control, established biofilms were exposed to ultrapure Milli-Q® water, which was used as the solvent for compound dilution. The resulting images were analyzed using COMSTAT2 software. *P. aeruginosa* H103 biofilms exhibited a flat and homogeneous architecture, with an approximate thickness of 16 μm (Fig. 3A). Upon squalamine and claramine A1 treatments, the residual biofilm appears to be sparse, indicating that both compounds induced a significant dispersion of pre-established biofilms of *P. aeruginosa* in this condition (Fig. 3A). COMSTAT2 analyses of CLSM images revealed a reduction of 45 ± 9.2 % or 60 ± 9.2 % of the biofilm biovolume after exposure to squalamine or claramine A1, respectively, as compared to the control condition (Fig. 3B). The biofilm average thickness was reduced by about 38 ± 7.5 % and 58 ± 7.6 % upon squalamine and claramine A1 exposure, respectively, and the maximal thickness was also reduced by about 27 % and 32 % for both compounds (Fig. 3B). Altogether, these data indicate that squalamine and claramine A1 can disperse a *P. aeruginosa* H103 pre-formed biofilm, with the dispersal effect being stronger in terms of biomass after exposure to claramine A1.

### 2.3 The spermine moiety of claramine A1 is involved in biofilm dispersion

**Fig. 4:**
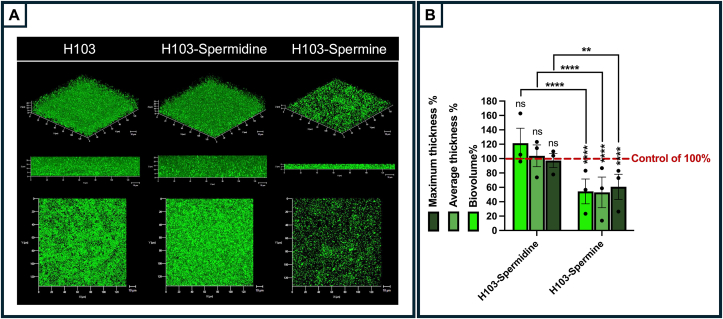
Spermine but not spermidine can disperse a 24 h-preformed biofilm of *P. aeruginosa* H103. (A) 3D shadow (upper panel), side (middle panel), and top (lower panel) CLSM images representative of a 24 h-old biofilm of *P. aeruginosa* after bacterial labeling with SYTO 9 (green) before (H103) and after 2 h of treatment with spermidine (H103-Spermidine) or spermine (H103-Spermine) at 1 μM concentration. Images show representative data from three independent biofilm assays. (B) COMSTAT2 analyses were performed to determine maximum thickness, average thickness and biovolume of the control condition (*P. aeruginosa* H103 treated with ultrapure Milli-Q® water for 2 h), and the assays (*P. aeruginosa* H103 upon spermidine or spermine treatment for 2 h). The control parameters were put at 100 % (red line). The error bars represent the standard error of the means (SEMs) and are the result of the analysis of at least four views of each of the three independent biological assays. Statistics were performed by ordinary one-way ANOVA followed by Tukey's multiple-comparison test. Values that are significantly different are indicated by asterisks as follows: ∗∗, *p* < 0,01; ∗∗∗∗, *p* < 0.0001; ns, not significant.

Squalamine and claramine A1 are aminosterols that primarily differ in the chemical groups at the C6 position. Squalamine contains a spermidine group, while claramine A1 features a spermine one(Fig. 1, [21]). We assessed if at least part of the dispersing activity could be related to the spermine and/or spermidine moieties. The effect of both polyamines was investigated by crystal violet staining in microtiter plates (Supplementary Fig. S4), or plates observed using CLSM (Fig. 4A) and COMSTAT2 analyses (Fig. 4B) after SYTO® 9 labeling. Exposure to spermine (1 μM) resulted in a sparse residual biofilm, indicating a significant dispersion of established *P. aeruginosa* biofilms. In contrast, spermidine at 1 μM did not induce the same effect (Fig. 4A), with a trend to promote biofilm formation, even if the increase was not significant (Fig. 4B). COMSTAT2 analyses of CLSM images revealed that spermine led to reduce significantly by about 45 %, 46 % and 36 %, the biofilm biovolume, average and maximal thicknesses, respectively, as compared to the control condition, while spermidine did not affect significantly neither of these structural biofilm parameters (Fig. 4B). Assays performed in microtiter plates (Supplementary Fig. S4) show that spermine can disperse a 24 h-old biofilm upon 2 or 24 h treatment, the effect being more pronounced in the latter case. Indeed, at 0.1 μM of spermine we observed about 34 % biofilm reduction after 24 h treatment compared to a biofilm reduction of about 20 % after 2 h exposition at the same concentration (Supplementary Fig. S4). By contrast, spermidine did not show any dispersion activity upon 24 h treatment, and about 20 % at the concentration of 0.1 μM. These data suggest that the spermine moiety of claramine A1 plays a key role in biofilm eradication, however, this effect is less pronounced for spermidine.

### 2.4 Spermine slightly increased the proportion of damaged cells within *P. aeruginosa* biofilm, while spermidine, squalamine and claramine A1 did not

Squalamine and claramine A1 at 1 μM did not affect *P. aeruginosa* growth in planktonic conditions (Supplementary Fig. S2). We then assessed their effect on biofilm-grown bacteria, using the fluorescent staining LIVE/DEAD™ BacLight™ Bacterial Viability Kit. In this assay, the intact viable cells were labeled in green using the SYTO 9 stain, and the permeabilized injured cells were labeled red, by the red propidium iodide. Neither squalamine nor claramine A1 at 1 μM led to alter the proportion of damaged cells relative to the alive cells within the biofilm, as compared to the control condition (Supplementary Fig. S5A). Upon spermine, but not spermidine treatment, we observed an increase of propidium iodide labeling (Supplementary Fig. S5B). In these conditions, spermine led to increase the proportion of damaged cells slightly but significantly within the biofilm by about 2.9-fold relatively to the alive cells, as compared to the control condition (Supplementary Fig. S5B).

### 2.5 Claramine A1 or spermine, but not squalamine or spermidine increased membrane fluidity

**Fig. 5:**
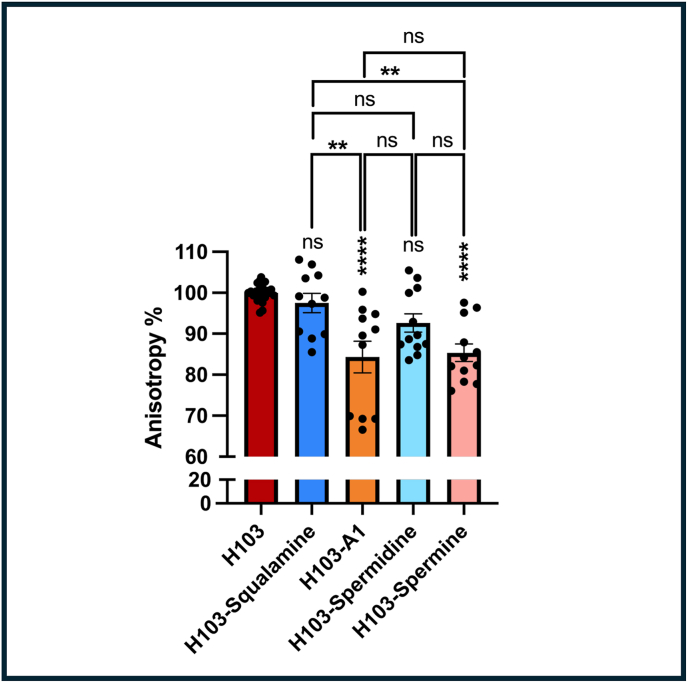
Claramine A1 and spermine treatments decreased *P. aeruginosa* H103 membrane fluidity. Membrane fluidity assessment by fluorescence anisotropy using 1,6-diphenyl-1,3,5-hexatriene (DPH) probe in *P. aeruginosa* H103 upon treatment with ultrapure Milli-Q® water (red bar), squalamine (blue bar), claramine A1 (orange bar), spermidine (light blue bar), or spermine (pink bar). Each experiment was assayed at least four times independently. Statistics were performed by ordinary one-way ANOVA followed by Tukey's multiple-comparison test. Significance was considered at ∗∗∗∗, *p* < 0.0001; ∗∗, *p* = 0.001–0.01; ns (not significant), *p* > 0.05.

Since squalamine and derivatives have been described as membrane active agents [21], we next assayed the membrane fluidity of *P. aeruginosa* in response to 1 μM of these compounds, by fluorescence anisotropy. To this end, we employed the 1,6-Diphenyl-1,3,5-hexatriene (DPH) fluorescent probe [25], which integrates into the hydrophobic regions of the lipid bilayer. A decrease in fluorescence anisotropy is linked to a reduction in the order of the phospholipid hydrocarbon chains, thereby indicating increased membrane fluidity [25]. Fluorescence anisotropy assays showed an enhancement of membrane fluidity when *P. aeruginosa* cells were exposed to 1 μM of claramine A1, but not after squalamine exposure (Fig. 5). In addition, a similar effect on *P. aeruginosa* membrane fluidity was observed when bacteria were treated with spermine but not with spermidine (Fig. 5). Such membrane fluidity variations can reflect the compounds integration within the bacterial membrane. It can be also the consequence of the bacterial response to a treatment. SigX is an extracytoplasmic sigma factor that responds to membrane stiffness [26,27] by increasing membrane fluidity *via* the production of short chain fatty acids [28]. We thus asked if *P. aeruginosa* may encounter a cell envelope stress in response to claramine A1 or spermine treatments. Total RNAs were then extracted and the RT-qPCR assays showed that while *sigX* gene itself was not affected in response to claramine A1 or spermine at 1 μM, its target gene *cmpX* [29] was slightly increased by about 1.5-fold upon claramine A1 treatment, but not spermine, suggesting that SigX activity was enhanced in response to claramine A1 (Supplementary Fig. S6)

### 2.6 Claramine A1, but not squalamine, nor spermine, nor spermidine, can potentialize the activity of doxycycline

Doxycycline is an antibiotic that belongs to the tetracyclines, to which *P. aeruginosa* is intrinsically resistant. Doxycycline was shown to synergize the activity of polymyxin B on multidrug resistant clinical *P. aeruginosa* strains [30]. Since claramine A1 and spermine perturbates *P. aeruginosa* membrane fluidity, and since claramine leads to increased SigX activity reflecting a cell envelope stress response, we asked if these compounds could potentiate the activity of doxycycline. Claramine A1 was previously shown to potentiate the activity of the tetracycline-like doxycycline antibiotic on *P. aeruginosa* ATCC27853 [21,31]. In a first step, we confirmed this effect on *P. aeruginosa* H103. The antimicrobial activities of the several compounds were determined by using a broth microdilution method performed in sterile 96-well microplates. After 24 h incubation, the minimal inhibitory concentration (MIC) was defined as the lowest concentration of the compound allowing no visible growth. In our conditions, the MIC of doxycycline against *P. aeruginosa* H103 was 16 μg mL^−1^. Adding claramine A1 at 1 μM led to reduce the MIC to 2 μg mL^−1^, while 1 μM squalamine was unable to synergize doxycycline. Interestingly, no effect was observed using 1 μM of spermine or spermidine, suggesting that the adjuvant activity of claramine A1 was not related to its spermine moiety alone (Supplementary Fig. S7).

### 2.7 Claramine A1 and spermine dispersal activity on a colistin resistant strain of *P. aeruginosa*

**Fig. 6:**
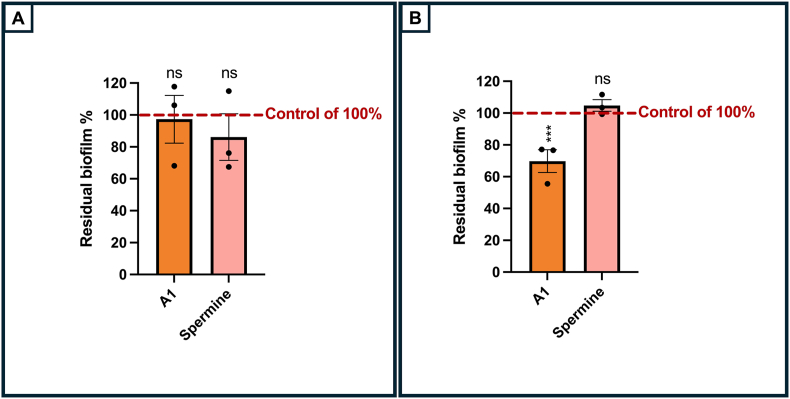
Biofilm dispersion of claramine A1 and spermine on *P. aeruginosa* strain 19.6947 (ColR) upon 2 h (A) or 24 h (B) treatment. Statistics were performed by unpaired *t*-test Significance was considered ∗∗∗, p = 0.0001–0.001; ns (not significant), p > 0.05.

As a cationic polyaminosterol, claramine A1 may exert its effects in a manner similar to that of the polymyxin colistin. We first assayed claramine A1 and spermine on a 24 h preformed biofilm of a colistin-resistant *P. aeruginosa* strain (ColR) 19.6947. As shown on Fig. 6, claramine A1 (1 μM) led to disperse a preformed *P. aeruginosa* ColR biofilm upon 24 h treatment by about 30 %, a level that was similar to the dispersion a preformed *P. aeruginosa* H103 biofilm (compare Fig. 6B and Supplementary Figure S4B to Fig. 2C). While claramine A1 was effective in dispersing *P. aeruginosa* H103 biofilm after 2 h of treatment, no such effect was observed in the colistin-resistant strain. No significant changes were noted after either 2 or 24 h of treatment. Spermine seems to disperse the biofilm of the ColR strain of *P. aeruginosa* upon 2 h exposure, however this dispersal effect is not significant. We then investigated whether spermine or claramine A1 could induce an adaptive mechanism of tolerance to colistin in *P. aeruginosa* by evaluating the transcription of the *arn* operon, which results in the addition of l-arabinose residues to lipopolysaccharide (LPS), altering its charge and reducing its interactions with cationic compounds. RT-qPCR assays revealed that the expression of the *arnA* gene was not significantly affected by claramine A1 or spermine at 1 μM, suggesting that the adaptive mechanism leading to polymyxin resistance was not primarily activated under our experimental conditions (Supplementary Fig. S6).

## 3 Discussion

Biofilms, which are structured communities of microbial cells encased in a self-produced polymeric matrix, pose significant challenges in clinical settings due to their resistance to conventional antibiotics and disinfectants. It is urgent to find alternative therapeutic strategies and new active molecules, particularly to counter chronic infections caused by bacterial species classified in the ESKAPE group since 2016 [32], and listed as critical and priority respectively, due to the emergence of strains resistant to conventional antibiotics [1,2]. Currently, no therapeutic agent has been identified that can successfully eradicate biofilms, and few innovative strategies targeting biofilms have emerged.

In this study, we explored the activity of the two aminosterols squalamine and claramine A1, against *P. aeruginosa* biofilms. Both compounds were previously shown to display a bactericidal activity against numerous Gram-positive and Gram-negative bacteria, including *P. aeruginosa* [15]. Herein, the MICs of squalamine and claramine A1 for *P. aeruginosa* H103 in our planktonic conditions were determined to be 16 μg mL^−1^, corresponding to 25.48 μM and 25.85 μM, respectively. These values are in a same range as those previously reported for *P. aeruginosa* PAO1 (MIC squalamine/MIC claramine A1, 16/8 μg mL^−1^), PA14 (16/8 μg mL^−1^), ATCC27853 (8/8 μg mL^−1^), or ATCC3337 (8/8 μg mL^−1^) [16,21]. According to the MICs data, at sub-MIC (0.001 μM–10 μM), both compounds showed limited impact on planktonic growth, except for a slight reduction with claramine A1 at 10 μM. The effects of squalamine and claramine A1 on *P. aeruginosa* biofilm formation showed that at certain sub-lethal concentrations, both compounds were able to prevent biofilm formation, with a more pronounced effect observed at 10 μM. Interestingly, we have recently demonstrated that squalamine and several 6-aminosterol derivatives can prevent biofilm formation of major resistant opportunistic pathogens, including carbapenem-resistant *P. aeruginosa* [23]. Noticeably, claramine A1 and squalamine possess a spermine or a spermidine moiety, respectively (Fig. 1, [16,21]). Interestingly, spermidine was shown to reduce biofilm formation in *E. coli* [33], and *Vibrio cholerae* [[34], [35], [36]], and spermine limited biofilm formation in *Neisseria gonorrhoeae* [37], or *V. cholerae* [38]. Altogether these data may suggest that these polyamine moieties may be involved at least partly in preventing biofilm formation in *P. aeruginosa*.

We also demonstrate that both squalamine and claramine A1 are capable of eradicating biofilm. The maximal biofilm dispersion was observed at 1 μM for both compounds after 2 h of treatment. This dispersal effect was confirmed through CLSM imaging and analysis, which showed that both claramine A1 and squalamine reduced biofilm biovolume, as well as the maximal and average thicknesses. Interestingly, claramine A1 caused a greater disruption of the biofilm, reducing biovolume and average thickness by 60 % and 58 % respectively, compared to reductions of 45 % and 38 % with squalamine, respectively. Altogether, these data show that both compounds can disperse an existing biofilm of *P. aeruginosa* at sub-MICs, without affecting the bacterial growth, suggesting that their use as adjuvants to conventional antibiotics activity. Indeed, eradication of biofilm infections is a major challenge because the biofilm phenotype provides bacteria with a protective environment from the immune system and antibiotics. Consequently, there is great interest in adjunctive molecules that cause biofilm dispersal. Although squalamine and claramine A1 do not affect bacterial growth under our conditions, and thus are unlikely to increase selection pressure, the potential risk of developing resistance and/or tolerance to these compounds should not be overlooked. Other agents were recently described with similar properties, such as the human atrial natriuretic hormone [39], osteocrine [40], sulfonamide derivatives [41], or matrix degrading enzymes [42,43]. In addition to their antibacterial activity on a large variety of Gram-positive and Gram-negative bacteria, and given their low cytotoxity against a large variety of host cells (CHO, HepG2, MDCK, 3T3, HaCat) with an IC_50_ values ranging from 70 to 86 μM [21], squalamine and claramine A1 could be very useful to tackle *P. aeruginosa* chronic infections.

As indicated earlier, squalamine and claramine A1 are aminosterols that possess water soluble groups, *i.e.* spermidine and sulfate in the case of squalamine, and spermine for claramine A1, and a lipophilic sterol core (Fig. 1, [16,21]). The bactericidal activity of squalamine on *P. aeruginosa* was attributed to its detergent-like mode of action and its ability to cause cytoplasmic membrane depolarization [16,17]. Noticeably, either the sterol, the spermidine chain, or the sulfate group, were shown to display only a low effect on their antimicrobial activity alone [18,19]. Membrane disruption was also shown upon claramine A1 exposure, at least on *Enterobacter aerogenes* EA289 and *S. aureus*. However, this polyaminosterol displayed also a peculiar mode of action through inhibition of the AcrAB-TolC efflux pump [21]. Claramine A1 dramatically disrupts the *Staphylococcus aureus* membrane, but no significant effect was found by using spermine alone [21], suggesting that the spermine moiety is not required for antibacterial activity. To understand the mode of action of squalamine and claramine A1 at sub-MIC on biofilm dispersion, we assessed the effects of their spermine and spermidine moieties on this phenotype. Remarkably, spermine at 1 μM concentration, but not spermidine led to eradicate *P. aeruginosa* biofilm, representing about 77 % of the claramine A1 activity, in our conditions. Moreover, spermidine seems to slightly promote biofilm in our conditions, according to the previous study of Hasan and coll. who showed that spermidine at 4 mM increased *P. aeruginosa* biofilm formation [44]. Altogether, these data show that the spermine moiety of claramine A1 is involved in biofilm eradication at 1 μM, whereas squalamine effect on biofilm eradication does not rely on its spermidine moiety, at least with the tested concentration. Accordingly, exogenously supplied spermidine did not mediate the disassembly of the preestablished biofilm of *E. coli or Salmonella enterica* [33], similarly to the data obtained herein. However, Spermidine was shown to drive *V. cholerae* dispersal *via* the transmembrane phosphodiesterase MbaA, and a polyamine importer PotD1 [45], suggesting a possible species-dependent effect of spermidine on biofilm eradication. Few data are reported concerning spermine. Spermine was shown not to cause dispersal of a preformed biofilm of *Neisseria gonorrhoeae* at 500 μM [37], or *V. cholerae in vitro* when used at concentrations of 100 μM [38]. However, in the study of Sobe et al., it was suggested that within the human intestine, the periplasmic solute binding protein NpsS could sense the exogenous spermine pools, which consequently relieves repression of the phosphodiesterase activity of MbaA, leading to degrading local c-di-GMP pools, and allowing the bacterial switch from sessile to planktonic lifestyle [38]. Noticeably, our assays were performed with 1 μM concentrations of each tested molecule, which is very low compared to the ones used in previous studies. Notably, both spermidine and spermine are abundant in the human intestine [46,47], with spermidine, which is nearly ubiquitous and self-produced by *P. aeruginosa* [48], detected at concentrations in the millimolar range. In contrast, spermine, which is typically found in eukaryotes, was detected at nearly 50 μM concentrations [49]. In the lungs, spermidine was quantified between 1 and 2 μM in a mouse model, and between 0.1 and 0.5 μM in a human host, except when treated with some antibiotics, where the concentration can reach 1 μM. In cystic fibrosis suffering patient, spermine concentration was evaluated at 3 μM [44].

Since claramine A1 and squalamine are aminosterol molecules known to be membrane-active [16,21], we further examined their effects on membrane fluidity using fluorescence anisotropy. Our results show that claramine A1, but not squalamine, increased *P. aeruginosa* membrane fluidity. One possible explanation for this could be a direct interaction between claramine A1 and the bacterial membrane, potentially linked to the spermine group, but not the spermidine group. Membrane fluidity was enhanced similarly as the whole claramine A1 compound upon spermine treatment, but not spermidine, suggesting that the spermine moiety is mainly involved in this phenotype. One of the differences between spermine and spermidine is the number of positive charges at physiological pH, respectively (Fig. 1). It is possible that these positive charges could interact with the negatively charged lipopolysaccharide layer to destabilize the outer membrane layer, similarly to polymyxins. Spermine possessing four net positive charges would thus interact more efficiently than spermidine with its three positive charges. In this regard, we show also that neither claramine A1 nor spermine affected the expression of the *arn* operon. Upregulation of this operon has been shown to result in the addition of l-arabinose residues to LPS, altering its overall negative charge to a neutral one, thereby reducing the electrostatic interactions between LPS and cationic compounds [50]. Such adaptative mechanism thus seems not to be activated in response to claramine A1 or spermine in our conditions. In addition, claramine A1 and to a lesser extend spermine, were active against a colistin-resistant *P. aeruginosa* strain suggesting that they could act in a different way than the cationic antimicrobials polymyxins. Another possibility is that claramine A1 and spermine would induce a cell envelope stress response [51], mediated by the ECF sigma factor SigX [26]. This ECF sigma factor is involved in *P. aeruginosa* membrane homeostasis [27]. Its activity triggers the production of small chain fatty acids, thus increasing membrane fluidity [28,52]. RT-qPCR analysis showed that SigX activity was only marginally increased upon claramine A1 treatment, a response that was not significant with spermine. This suggests that the SigX-mediated mechanism is likely not primarily involved under our experimental conditions.

This study highlights the potential of squalamine, claramine A1, and spermine as *P. aeruginosa* biofilm-dispersing adjuvants of conventional antibiotics that can be used at sub-inhibitory concentrations, offering a promising approach to combat biofilm-related infections. However, even if limited, the risk that these compounds could allow dissemination of planktonic bacteria involved in acute infections, even in presence of antibiotics, cannot be neglected. The ability to disrupt biofilms without significant bactericidal activity may help reduce resistance development. While claramine A1 is easy to synthesise [21] compared to squalamine, these findings support further exploration of aminosterol derivatives and their combination with conventional antibiotics or phages to treat chronic infections caused by *P. aeruginosa*. Further studies on the molecular mechanisms involved are needed to better understand the differences between squalamine and claramine A1, and to advance these compounds—particularly claramine A1—toward clinical applications.

## 4 Experimental procedures

### 4.1 Bacterial strains, growth conditions and chemicals

*P. aeruginosa* H103, a prototroph of the wild-type strain PAO1 from R.E.W. Hancock's Lab [53], and *P. aeruginosa* colistin-resistant clinical 19.6947 strain (French national reference center for antibiotic resistance, Besançon), were grown in Luria-Bertani broth containing 171 mM (10 g L^−1^) NaCl (LB, Difco, BD). *P. aeruginosa* strains were grown overnight (18 h) in LB at 37 °C with shaking (180 rpm). Then a bacterial suspension at A_580 nm_ of 0.1 was grown in LB for 24 h at 37 °C with or without shaking (180 rpm). Squalamine and claramine A1, synthesized as previously described [21], and spermine (S4264, Sigma-Aldrich) and spermidine (S2626, Sigma-Aldrich) were solubilized in ultrapure Milli-Q® water at 10 mM, and kept at −20 °C. In the same way, a stock solution of antibiotic doxycycline (D9891, Sigma-Aldrich) was solubilized in ultrapure Milli-Q® water at 4 mg mL^−1^, and kept at −20 °C for 48 h. Each set of experiments was performed at least three times.

### 4.2 Bacterial growth monitoring

To assess the effect of squalamine and claramine A1 on *P. aeruginosa* H103 growth kinetics, bacterial cultures were grown in the presence or absence of the compounds at concentrations ranging between 0.001 and 10 μM. An absorbance measure at 580 nm was performed every 30 min using the Spark 20 M multimode Microplate Reader controlled by Spark Control™ software Version 2.1 (Tecan Group Ltd., Crailsheim, Germany). The data were plotted, and each point indicates the mean ± standard error of the mean (SEM) of A_580nm_ values. Each set of experiments was performed at least three times.

### 4.3 Determination of minimal inhibitory concentrations

The antimicrobial activity of the compounds was assayed by using a broth microdilution method performed in sterile 96-well microplates (Thermo Fisher Scientific, NuncTM, Waltham, MA, USA), according to the European Committee on Antimicrobial Susceptibility Testing (EUCAST) standards with some modifications. Briefly, *P. aeruginosa* H103 was grown in LB overnight, and adjusted to 5.10^5^ CFU mL^−1^ in LB, before being exposed to squalamine or claramine A1 at concentrations ranging between 32 and 0.0625 μg mL^−1^, or to doxycycline between 128 and 0.5 μg mL^−1^. Growth was performed at 37 °C for 24 h without shaking, and the minimal inhibitory concentration (MIC) was defined for each compound from at least triplicate observations as the lowest concentration of the compound allowing no visible growth. Bacterial viability was assessed by adding resazurin (Acros-organics) at a final concentration of 0.0125 mg ml^−1^ to the microplate wells. After incubation for 2 h at 37 °C, the colour of the wells was observed. A pink or purple colour change reflected active cell metabolism, whereas an unchanged colour (blue) reflected the MIC. The adjuvant effect of squalamine, A1, spermidine and spermine on doxycycline was also studied. Briefly, the MIC was determined for the combination of the compounds concentrated to 1 μM with a concentration range of doxycycline from 16 to 1 μg ml^−1^. Bacterial viability was also assessed by using resazurin.

### 4.4 Pyocyanin quantification assay

*P. aeruginosa* H103 untreated or treated with squalamine or claramine A1 at a concentration of 1 μM was grown in LB in a 96-well microtiter plate (Thermo Fisher Scientific, Nunc™, Waltham, MA, USA) at 37 °C for 24 h with shaking (180 rpm). After incubation for 24 h, A_580 nm_ was measured to determine the bacterial growth. The pyocyanin quantification assay was carried out as previously described [54,55]. Briefly, one volume of chloroform was added to the culture supernatants. After centrifugation, the chloroform blue layer was collected and ½ volume of 0.5 M HCl was added. After a second centrifugation, the red pink HCl layer was collected and the A_520 nm_ was recorded (Spark 20 M multimode Microplate Reader, Tecan Group Ltd., Crailsheim, Germany). The data were normalized to the bacterial cell density (A_520 nm_/A_580 nm_) and expressed as a percentage of the control value.

### 4.5 Membrane fluidity evaluation by fluorescence anisotropy

Membrane fluidity of *P. aeruginosa* H103 untreated and treated cultures with 1 μM of squalamine or claramine A1 was performed by fluorescence anisotropy as previously described [56]. Briefly, bacteria (A_580nm_ = 0.1) were grown for 24 h with shaking (180 rpm) in 96-well microtiter plates, and 2 μL out of 200 μL of squalamine, or claramine A1 (1 μM final concentration), or ultrapure Milli-Q® water (control condition), were added for 2 h. Bacterial cells were centrifuged (7500 *g* for 5 min at room temperature). Pellets were washed twice with 10 mM MgSO_4_.7H_2_O before resuspension in the same wash solution to reach an A_580 nm_ of 0.1. Then, 1 μL of 4 mM 1,6-diphenyl-1,3,5-hexatriene (DPH) dissolved in tetrahydrofuran was added to 1 mL aliquot of the diluted suspension and incubated at 37 °C for 30 min in the dark to allow the probe to incorporate into the bacterial membranes. Fluorescence polarization was measured using the Spark 20 M multimode Microplate Reader, equipped with an active temperature regulation system (Spark 20 M multimode Microplate Reader, Tecan Group Ltd., Crailsheim, Germany), with wavelengths of emission and excitation set at 425 nm and 365 nm, respectively. The results are expressed as a percentage of anisotropy compared with the control.

### 4.6 Biofilm prevention and eradication assays

Biofilm formation was performed in 96-well microtiter plates (Thermo Fisher Scientific, Nunc™, Waltham, MA, USA). After overnight growth, a culture of *P. aeruginosa* H103 was adjusted at A580 of 0.1 in LB. Squalamine and claramine A1 at various concentrations were added at the same time as the inoculum. The biofilms were incubated under static conditions at 37 °C for 24 h, and A_580nm_ was recorded. Biofilm was quantified by discarding the medium, rinsing the wells with distilled water, and staining bounded bacteria with crystal violet at 0.1 % for 15 min. The dye was then solubilized in 30 % v/v acetic acid and A_595 nm_ was recorded using the Spark 20 M multimode microplate reader controlled by Spark Control™ software Version 2.1 (Tecan Group Ltd., Crailsheim, Germany). In each experiment, background staining was adjusted by subtracting the crystal violet bound to uninoculated control wells. Data were normalized to the bacterial density (A_595 nm_/A_580 nm_) and expressed as a percentage of the control value. Biofilm eradication was assessed as previously described [23] with the following changes. *P. aeruginosa* H103 overnight culture was adjusted to A_580 nm_ of 0.1 in LB, and the biofilm was grown in static conditions at 37 °C on pegs displayed on the cover of microtiter plates (Nunc™ Immuno TSP Lids). The pegs were rinsed with physiological water (0.09 % NaCl) before being treated with squalamine, claramine A1, spermine or spermidine at various concentrations, or with ultrapure Milli-Q® water (control) for 2h or 24h at 37 °C in static condition. The pegs were then rinsed three times with distilled water and stained with 0.1 % crystal violet for 15 min at room temperature. The dye was then detached with 30 % acid acetic and the A_595 nm_ was determined using the Spark 20 M multimode Microplate Reader. The data were expressed as a percentage of the control value.

### 4.7 Biofilm dispersion assays and confocal laser scanning microscope analysis (CLSM)

An overnight culture of *P. aeruginosa* H103 was adjusted to an A_580 nm_ of 0.1, added to a 24-well glass-bottom plate or Petri dish containing two cover glasses, and incubated at 37 °C in static conditions. After 24 h, residual planktonic bacteria were removed, and the biofilm formed on the glass surface was treated for 2 h with 1 μM of claramine A1, squalamine, spermine or spermidine. The residual biofilm was then rinsed twice with physiological water, labeled with fluorescent dyes, and observed by CLSM. Biofilm cells were stained by adding 5 μM of SYTO 9 green fluorescent nucleic acid stain (Invitrogen, Thermo Fisher Scientific; excitation at 488 nm and emission from 500 to 550 nm). The live/dead fluorescent labelling was performed using the LIVE/DEAD BacLight Bacterial Viability Kit (Thermo Fisher). Cells were labeled with a mixture (vol/vol) of SYTO 9 (3,34 mM) and propidium iodide (20 mM) according to the recommendations of the supplier. The CLSM observations were carried out with a Zeiss LSM710 (Carl Zeiss Microscopy, Oberkochen, Germany) using a 40× oil immersion objective. Images were taken every 0.5 μm throughout the whole biofilm depth. For visualization and processing of three-dimensional (3D) images, the Zen 2.1 SP1 zen software (https://www.zeiss.com/microscopy/int/downloads/zen.html) (Carl Zeiss Microscopy, Oberkochen, Germany) was used. The average and maximum thicknesses (μm) and biovolumes (μm^3^.μm^−2^) of biofilms were measured using the COMSTAT software [57] (http://www.imageanalysis.dk/). At least five image stacks from each of the three independent experiments (fifteen stacks in total) were used for each analysis. At least five image stacks from each of the six independent experiments (thirty stacks in total) were used for analysis of biofilm treated with squalamine and claramine A1. For analysis of biofilm treated with spermidine and spermine, at least four image stacks from each of the three independent experiments (twelve stacks in total) were used.

### 4.8 RNA extraction and quantitative RT-PCR

An overnight culture of *P. aeruginosa* H103 was adjusted to an A_580 nm_ of 0.1, and allowed to grow in LB for 24 h before claramine A1, or spermine at 1 μM was added for 2 h. Total RNAs from three independent cultures were isolated by the hot acid-phenol method as previously described [58], with minor modifications. After rigorous treatments with the Turbo DNAse™ kit (Invitrogen), according to the manufacturer, the removal of DNA contaminants was verified by PCR amplification. RNAs were then quantified, and their quality was checked by electrophoretic migration on a 2 % agarose gel. Synthesis of cDNA and RT-qPCR was achieved as previously described [59], using the oligonucleotides listed in Supplementary Table S1. The expression level of mRNAs was calculated by comparing the threshold cycles (Ct) of target genes with those of the control group, and the relative quantification was determined with the 2^−ΔΔCt^ method [60] using DataAssist™ software (Applied Biosystems).

### 4.9 Statistical analyses

All experiments were carried out at least three times independently and the mean with standard error of the mean (SEM) was calculated and plotted. Each replicate was represented. Statistical significance was evaluated using Prism GraphPad software version 10.4.1. The data were statistically analyzed using unpaired *t*-test or ordinary one-way analysis of variance (ANOVA) followed by Dunnett or Tukey multiple comparison test to calculate *p* values. Significance was considered at ∗∗∗∗, *p* < 0.0001; ∗∗∗, *p* = 0.0001–0.001; ∗∗, *p* = 0.001–0.01; ∗, *p* = 0.01–0.05; ns (not significant), p > 0.05.

## CRediT authorship contribution statement

**Anne-Sophie Tareau:** Writing – review & editing, Methodology, Investigation, Formal analysis, Data curation. **Ali Tahrioui:** Writing – review & editing, Validation, Methodology, Formal analysis. **Mathieu Gonzalez:** Formal analysis, Methodology. **Evan Croize:** Methodology, Formal analysis. **Jennifer Varin Simon:** Writing – review & editing, Methodology, Formal analysis. **Magalie Barreau:** Methodology, Formal analysis. **Audrey David:** Writing – review & editing, Methodology, Formal analysis. **Fany Reffuveille:** Writing – review & editing, Supervision. **Jean-Michel Brunel:** Writing – review & editing, Supervision, Resources, Project administration. **Olivier Lesouhaitier:** Writing – review & editing, Writing – original draft, Validation, Supervision, Resources, Project administration, Investigation, Funding acquisition, Formal analysis, Data curation, Conceptualization. **Sylvie Chevalier:** Writing – review & editing, Writing – original draft, Validation, Supervision, Resources, Project administration, Investigation, Funding acquisition, Formal analysis, Data curation, Conceptualization.

## Declaration of competing interest

The authors declare that they have no known competing financial interests or personal relationships that could have appeared to influence the work reported in this paper.

## Data Availability

No data was used for the research described in the article.
