# Supplementary material for: Squalamine and claramine A1 disperse Pseudomonas aeruginosa biofilm
Source: Biofilm. 2025 May 27;9:100293. doi: 10.1016/j.bioflm.2025.100293 (PMC12166708; doi:10.1016/j.bioflm.2025.100293)

**Squalamine and Claramine A1 disperse *Pseudomonas aeruginosa* biofilm**


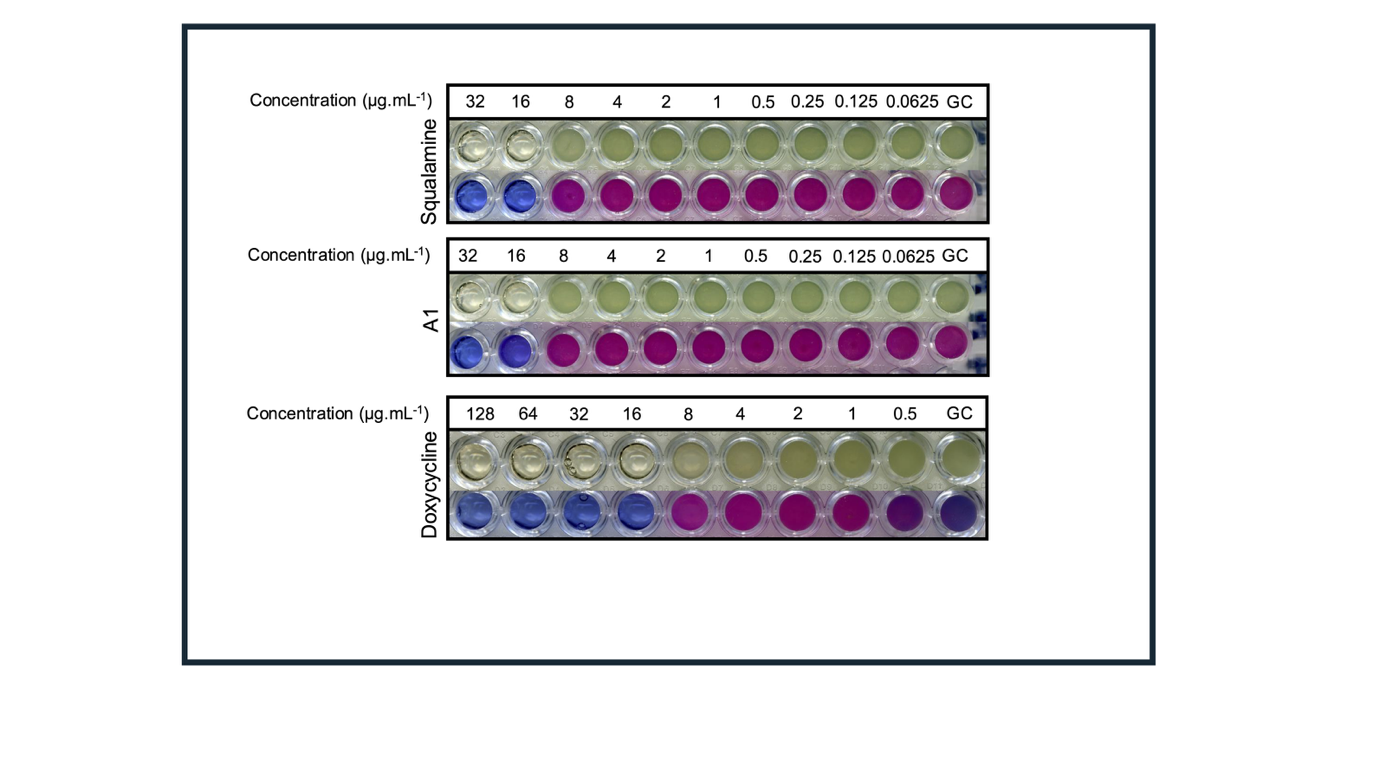


**Supplementary Figure S1.** Minimal inhibitory concentrations (MIC) of squalamine, claramine A1 and doxycycline against *P. aeruginosa* H103. Bacterial growth was observed in absence (upper line) or in presence of resazurin (lower line) to ascertain bacterial viability. Pink or purple color is the reflect of metabolically active cells. Blue color is the reflect of metabolically inactive or dead cells. GC: Growth control.


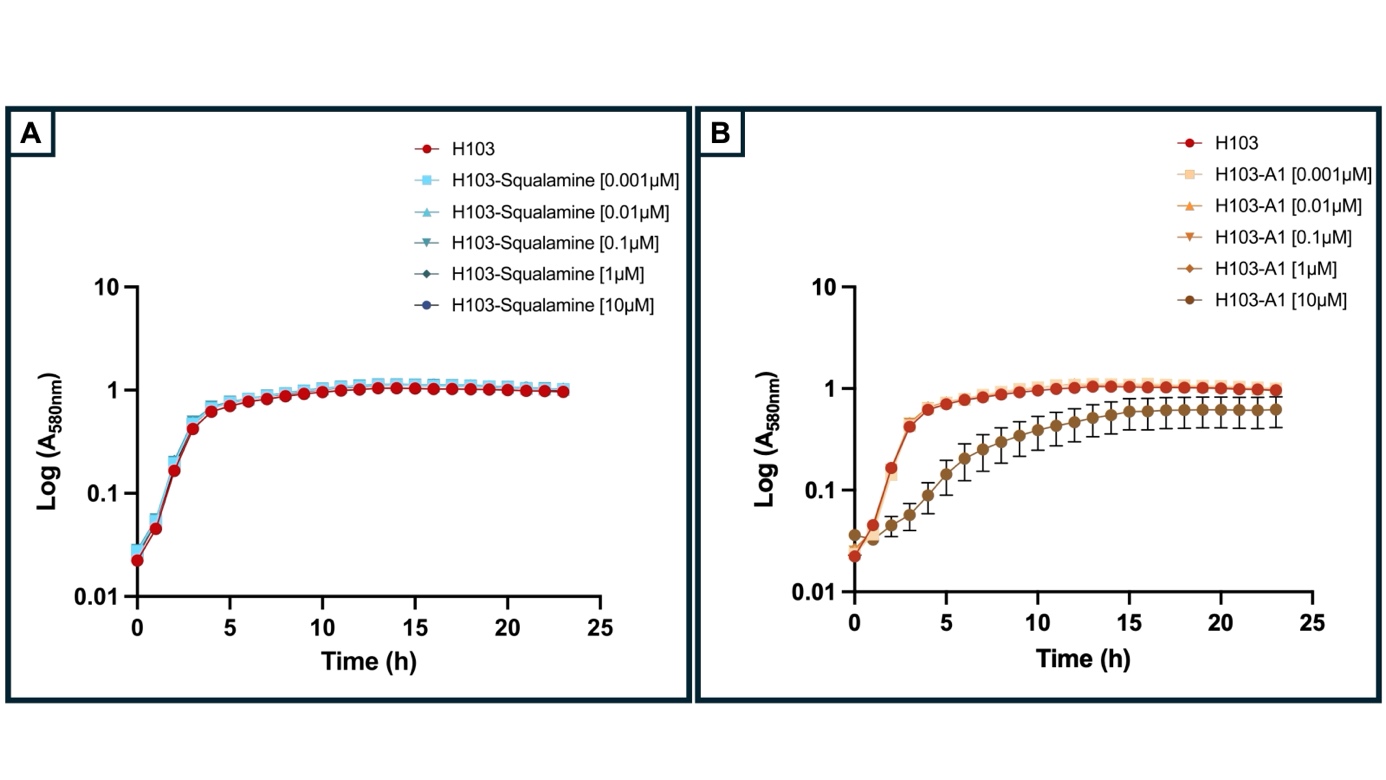


**Supplementary Figure S2.** Growth kinetics of *P. aeruginosa* H103 in presence of various concentrations of squalamine (A) or claramine A1 (B) ranging from 0.001 to 10 µM. The error bars represent the standard error of the means (SEMs) and are the result of the analysis of three independent biological assays.


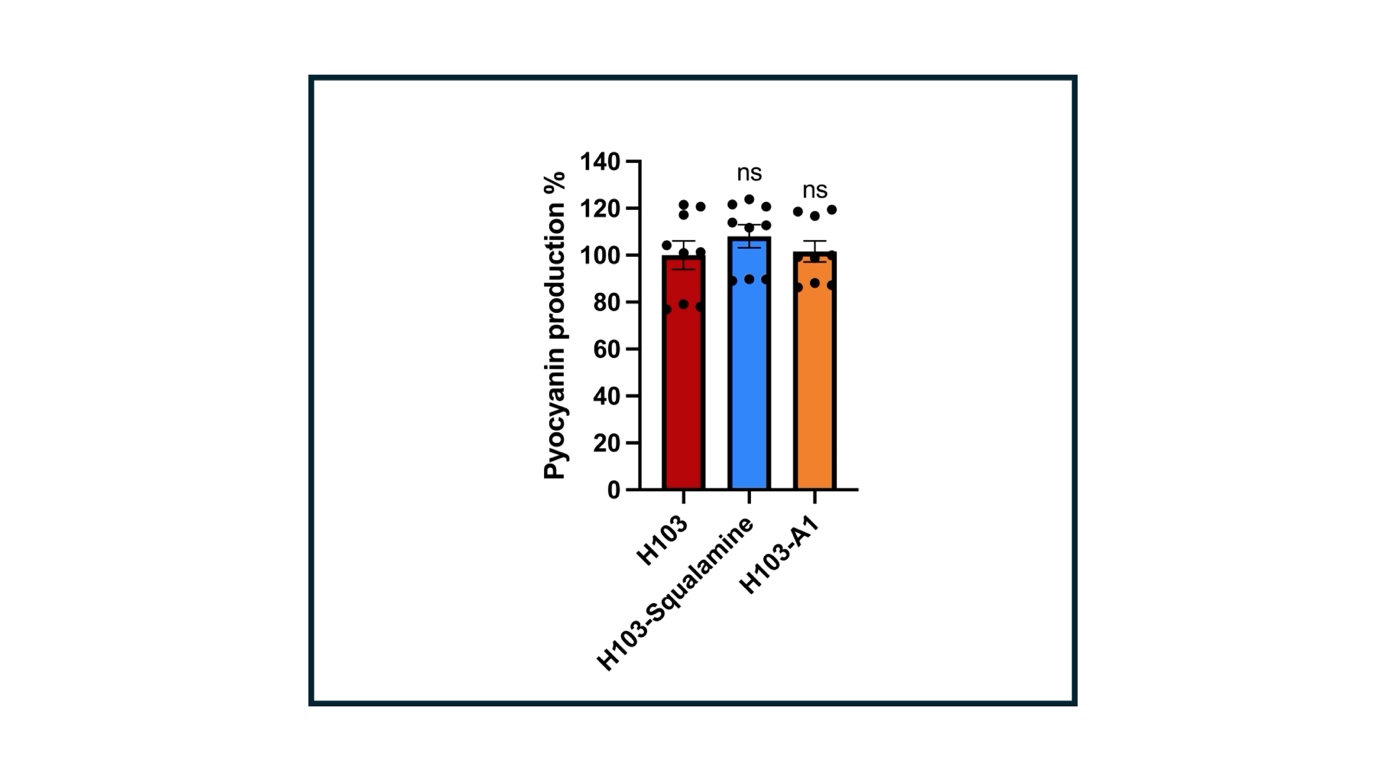


**Supplementary Figure S3.** Neither squalamine, nor claramine A1 modulate the production of pyocyanin. Pyocyanin was extracted as described in the materials and methods section, from *P. aeruginosa* H103 grown in presence of water (control, red bar), squalamine (blue bar), or claramine A1 (orange bar) at 1 µM. Statistics were performed by ordinary one‐way ANOVA followed by Tukey's multiple‐comparison test. ns, not significant.


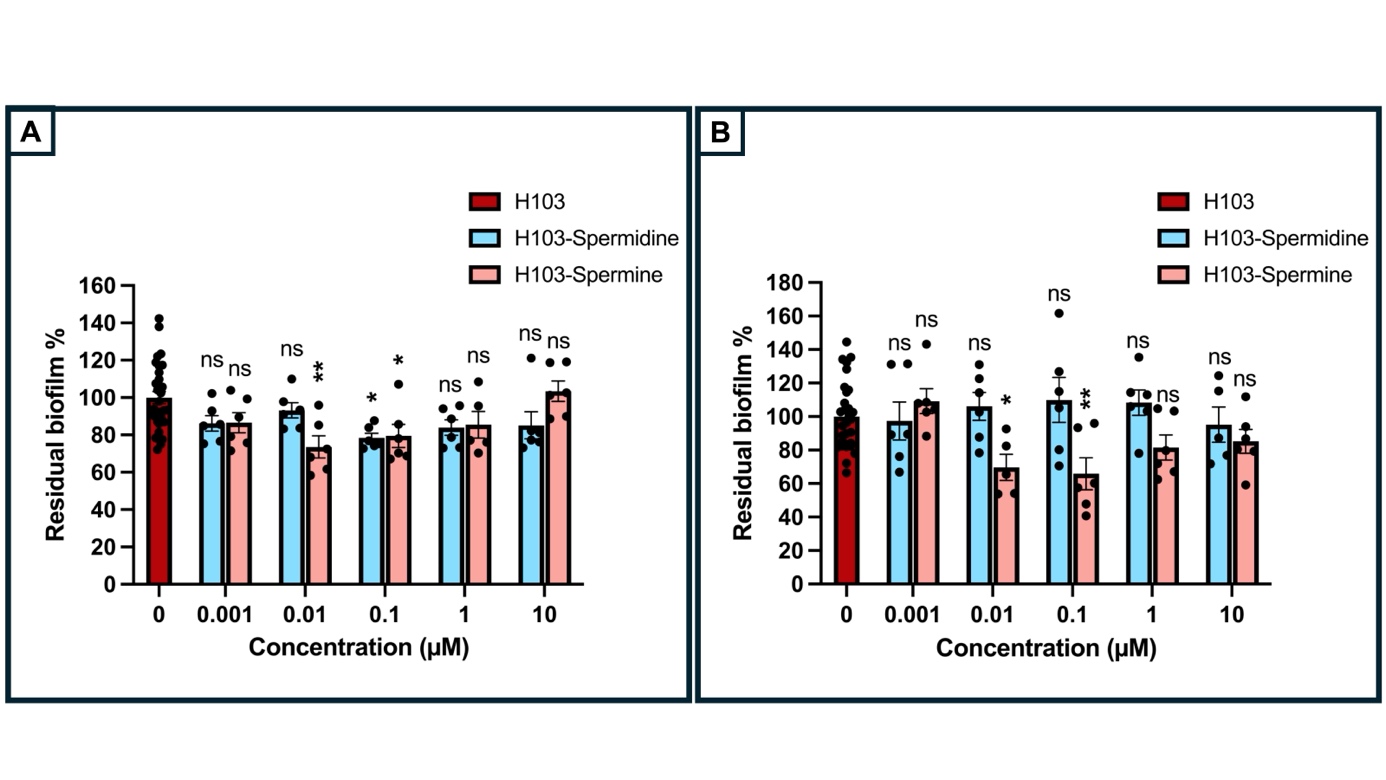


**Supplementary Figure S4**. Spermine (pink bars) and to lesser extent spermidine (blue bars) can eradicate a 24 h-preformed biofilm upon 2 h (A) or 24 h (B) treatments at different concentrations. Statistics were performed by ordinary one‐way ANOVA followed by Tukey’s multiple‐comparison test. Significance was considered **, *p* = 0.001–0.01; *, *p* = 0.01–0.05; ns (not significant), *p* > 0.05.

**
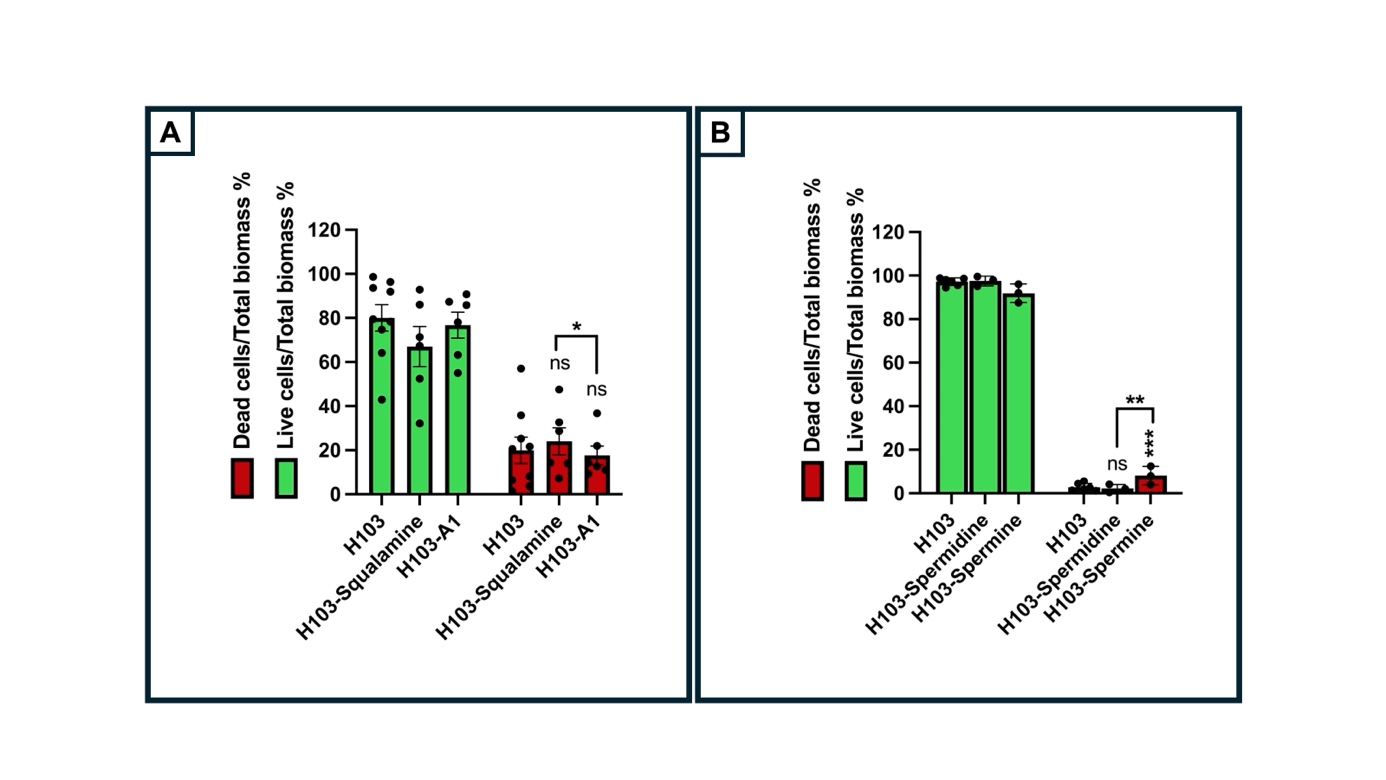
**

**Supplementary Figure S5**. COMSTAT2 analyses of CLSM images of 24 h old biofilms of *P. aeruginosa* H103 exposed for 2 h to (A) squalamine (H103-Squalamine), claramine A1 (H103-A1), or water (H103), or (B) to spermidine (H103-Spermidine), spermine (H103-Spermine) or not (H103), after labelling by SYTO 9 (green) and propidium iodide (red). COMSTAT2 analyses were performed to determine biovolume of live cells (green) or injured cells (red). The error bars represent the standard error of the means (SEMs) and are the result of the analysis of a minimum of four views of each of the six (A), or three (B) independent biological assays. Statistics were performed by ordinary one‐way ANOVA followed by Tukey's multiple‐comparison test. Significance was considered at ***, *p* = 0.0001–0.001; **, *p* = 0.001–0.01; *, *p* = 0.01–0.05; ns (not significant), *p* > 0.05.

**
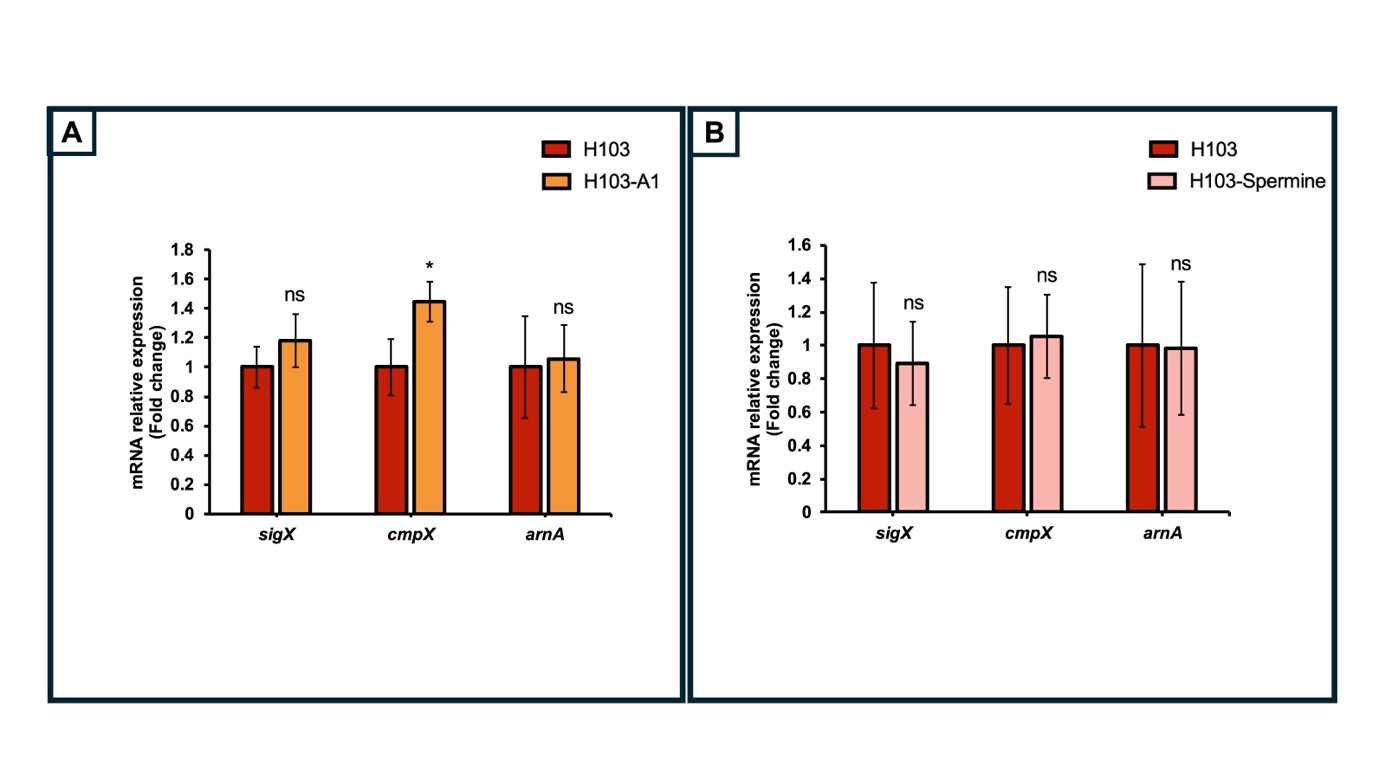
**

**Supplementary Figure S6.** Evaluation by RT-qPCR of *P. aeruginosa* gene expression upon exposure to 1 µM of claramine A1 (H103-A1) or spermine (H103-spermine) or water (H103). Statistics were performed by unpaired t-test. Significance was considered at *, *p* = 0.01–0.05; ns (not significant), *p* > 0.05.

**
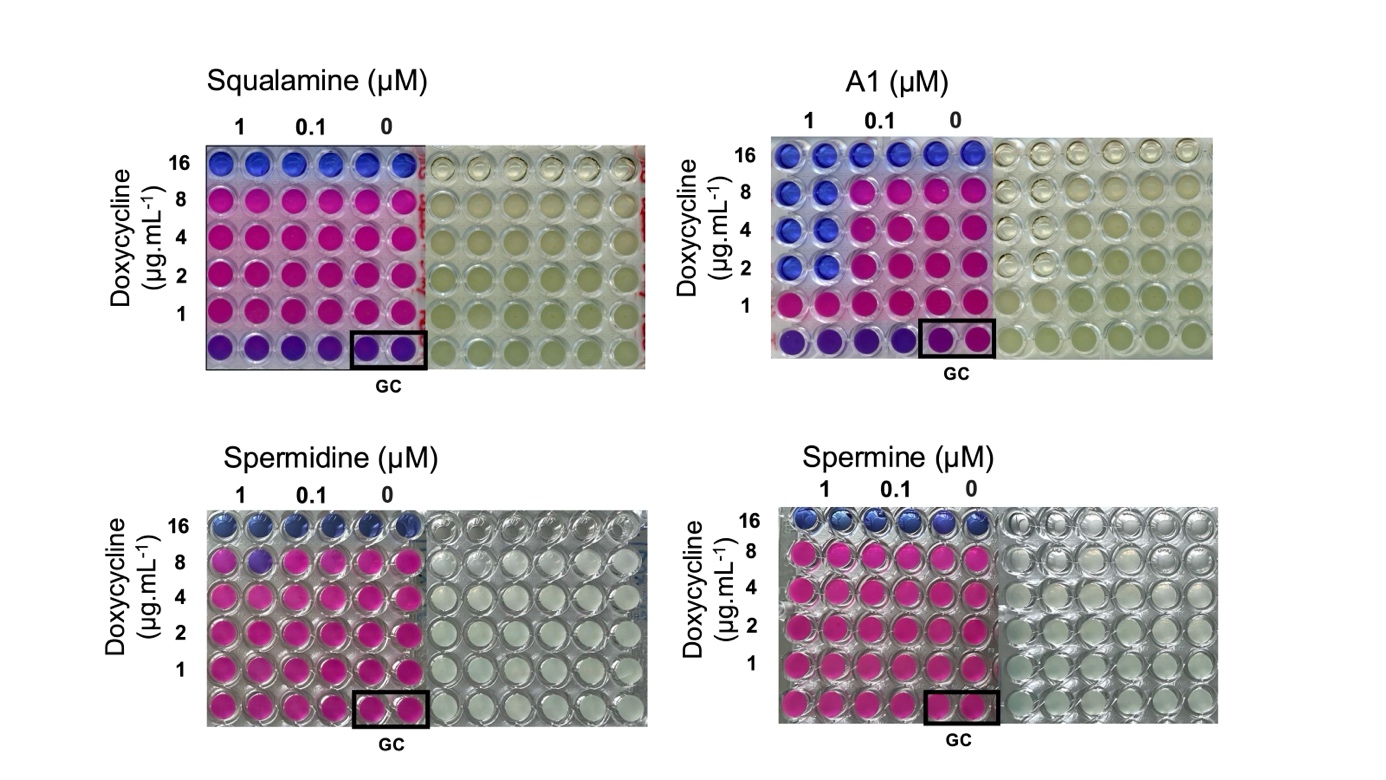
**

**Supplementary Figure S7**. Claramine A1 can synergyse doxycycline. Combinations between claramine A1, squalamine, spermine, or spermidine (1 or 0.1 µM), and doxycycline at concentrations ranging from 0 to 16 µg.mL^-1^ were assayed on *P. aeruginosa* H103 grown into microtiter plates for 24h, before being treated with resazurin to ascertain bacterial viability (left side of the microtiter plate), or not (right side of the microtiter plate). Blue color is the reflect of metabolically inactive or dead cells. GC : Growth control.

**Supplementary Table S1.** List of the primers used for RT-qPCR experiments.


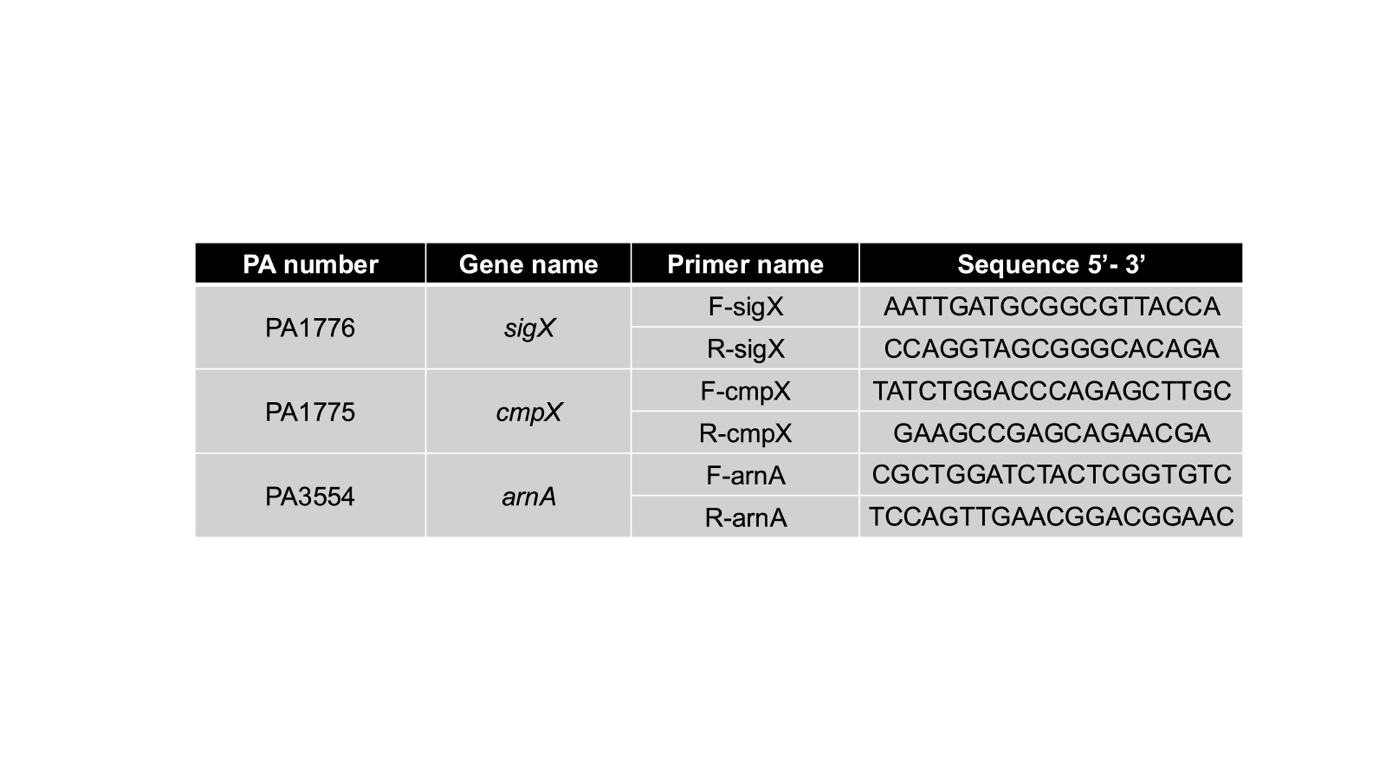

Supplement: Multimedia component 1 [file mmc1.docx]
